# Effect of stem cell transplantation on patients with ischemic heart failure: a systematic review and meta-analysis of randomized controlled trials

**DOI:** 10.1186/s13287-019-1214-0

**Published:** 2019-04-18

**Authors:** Yixuan Wang, Fen Xu, Jingwei Ma, Jiawei Shi, Si Chen, Zongtao Liu, Junwei Liu

**Affiliations:** 1 Department of Cardiovascular Surgery, Union Hospital, Tongji Medical College, Huazhong University of Science and Technology, Wuhan, 430022 Hubei China; 2 Department of Biochemistry and Molecular Biology, Tongji Medical College, Huazhong University of Science and Technology, Wuhan, 430030 Hubei China

**Keywords:** Stem cell transplantation, Ischemic heart failure, Meta-analysis

## Abstract

Stem cell transplantation (SCT) has become a promising way to treat ischemic heart failure (IHF). We performed a large-scale meta-analysis of randomized clinical trials to investigate the efficacy and safety of SCT in IHF patients. Randomized controlled trials (RCTs) involving stem cell transplantation for the treatment of IHF were identified by searching the PubMed, EMBASE, SpringerLink, Web of Science, and Cochrane Systematic Review databases as well as from reviews and the reference lists of relevant articles. Fourteen eligible randomized controlled trials were included in this study, for a total of 669 IHF patients, of which 380 patients were treated with SCT. The weighted mean difference (WMD) was calculated for changes in the New York Heart Association (NYHA) class, left ventricular ejection fraction (LVEF), left ventricular end-diastolic and end-systolic volumes (LVEDV and LVESV), and Canadian Cardiovascular Society (CCS) angina grade using a fixed effects model, while relative risk (RR) was used for mortality. Compared with the control group, SCT significantly lowered the NYHA class (MD = − 0.73, 95% CI − 1.32 to − 0.14, *P* < 0.05), LVESV (MD = − 14.80, 95% CI − 20.88 to − 8.73, *P* < 0.05), and CCS grade (MD = − 0.81, 95% CI − 1.45 to − 0.17, *P* < 0.05). Additionally, SCT increased LVEF (MD = 6.55, 95% CI 5.93 to 7.16, *P* < 0.05). However, LVEDV (MD = − 0.33, 95% CI − 1.09 to 0.44, *P* > 0.05) and mortality (RR = 0.86, 95% CI 0.45 to 1.66, *P* > 0.05) did not differ between the two groups. This meta-analysis suggests that SCT may contribute to the improvement of LVEF, as well as the reduction of the NYHA class, CCS grade, and LVESV. In addition, SCT does not affect mortality.

**Electronic supplementary material:**

The online version of this article (10.1186/s13287-019-1214-0) contains supplementary material, which is available to authorized users.

## Background

Ischemic heart disease (IHD), one of the leading causes of morbidity and mortality around the world, occurs when the myocardial oxygen supply cannot meet the myocardial oxygen demand [1, 2]. Revascularization to revive stunned or hibernating myocardium is beneficial for IHD patients, but the ventricular remodeling process is usually irreversible, particularly in end-stage IHD patients [3]. When IHD reaches an advanced stage, further revascularization and medical therapy may be useless [3–6]. The injured myocardium often disappears and is replaced by scar tissue, thereby resulting in systolic dysfunction, myocardial remodeling, and finally, heart failure [5, 7]. Therefore, a new therapeutic strategy is urgently needed to break through these limitations.

Since its discovery, stem cell transplantation (SCT) has become a new treatment strategy to improve cardiac function in patients with advanced ischemic heart failure [8]. SCT functions to enhance tissue perfusion contribute to new blood vessel growth and preserve or even regenerate myocardial tissue [9–11]. The first case of applying SCT to treat myocardial infarction occurred in 2001, which ushered in the clinical trials of utilizing stem cell transplantation to treat ischemic heart failure (IHF). Since then, more clinical studies have focused on this method and indicated that stem cells are safe, exhibit few treatment-related adverse events, and do not increase the incidence of major adverse cardiac events in comparison with control groups [11–14].

Pre-clinical trials have shown that different types of SCT facilitate graft survival and the formation of new contractile tissue [15]. Among them, stem cells such as autologous bone marrow mononuclear cells (BMMNCs) are especially widely used by many clinicians as a form of SCT to treat IHF [6, 14, 16–18]. Although several studies have investigated the effects of SCT on IHF, the efficacy and side effects of intramyocardial stem cell injection still remain unknown [6, 12–14]. A better understanding of the role of SCT in IHF is crucial in deciding whether SCT should be implemented. Therefore, we conducted a systematic review and meta-analysis of randomized clinical trials to evaluate the effects of intramyocardial stem cell injection and other forms of SCT on myocardial repair in ischemic heart failure.

## Materials and methods

### Search strategy

A systematic literature search was conducted using the PubMed, EMBASE, SpringerLink, Web of Science, and Cochrane Systematic Review databases to screen and identify all eligible studies published up to August 8, 2018, and restricted to English-language literature. The following free-text search terms and Medical Subject Headings for patients with heart failure were used during the search when applicable: ischemic heart failure, stem cell transplantation, randomized controlled trials. This comprehensive meta-analysis was conducted strictly in accordance with the Preferred Reporting Items for Systematic Reviews and Meta-Analyses of individual participant data (PRISMA-IPD) statement [19]. The PRISMA checklist is shown in Additional file 1: Figure S1. The authors were contacted when the methodology of the clinical trial or the results were not clear or when relevant data were not reported. In the case of a discrepancy, another investigator was consulted to reach an agreement.

### Study selection

Two experienced investigators blindly performed the study selection strictly in conformity with the population, intervention, comparison, and outcomes (PICO) principle, and disagreements were settled by discussion with any of the other investigators in this paper. Clinical trials were eligible for inclusion if (i) the participants were diagnosed with heart failure of an ischemic origin, (ii) they were randomized clinical trials that compared the use of SCT with a placebo, and (iii) outcome indicators were as follows: primary indicators included New York Heart Association (NYHA) class and mortality. Secondary indicator was left ventricular ejection fraction (LVEF), Canadian Cardiovascular Society (CCS) angina grade, left ventricular end-diastolic volume (LVEDV), and left ventricular end-systolic volume (LVESV).

Studies were excluded if they (i) were not randomized controlled trials, (ii) were duplicate publications, (iii) lacked the targeted indicators, (iv) had the design and rationale of a preconceived trial, and (v) were published as a conference proceeding.

### Data extraction

Eligible studies were independently reviewed by two investigators (Yixuan Wang and Jingwei Ma), and disagreements were resolved through a discussion with a third reviewer (Junwei Liu). The following information was extracted from the included studies using a predefined form: first author, year of publication, sample size, age range, country, sex, cell type, study design, number of participants in the experimental and control groups, follow-up duration, patient baseline characteristics, intervention, and main outcome measures.

### Quality assessment

A methodological quality assessment was conducted independently by two investigators (Fen Xu and Zongtao Liu) using the Cochrane criteria. The items used for the assessment of each study included selection bias (random sequence generation, allocation concealment), performance bias (blinding of participants and personnel), detection bias (blinding of outcome assessment), attrition bias (incomplete outcome data), reporting bias (selective reporting), and other bias. We judged the studies “yes” to show a low risk of bias, “no” to show a high risk of bias, and “unclear” to indicate an unclear or unknown risk of bias using the recommendations of the Cochrane Handbook.

### Data analysis

Data analyses were conducted using the Review Manager software (version 5.3). All outcomes contained at least three trials for the meta-analysis. The change from baseline to post-stem cell transplantation between each group was calculated. Relative risk (RR) with a 95% confidence interval (95% CI) was used to express dichotomous data, and the weighted mean difference (WMD) with standard deviation (SD) was used to express continuous data. We measured weighted *I*^2^ to assess both the interrater variability for study inclusion and the methodological quality [20]. An *I*^2^ score of < 50% indicated moderate heterogeneity, while any score > 50% was considered to be extensively heterogeneous. When moderate heterogeneity was present, we used the Mantel-Haenszel (M-H) fixed effects model to conduct the meta-analysis; if not, we used a random effects model [21]. Heterogeneity between studies was assessed using the *I*^2^ statistic, its 95% confidence interval, and the Cochrane *Q* test. Publication bias was assessed using a funnel plot. A *P* value less than 0.05 was considered statistically significant in the meta-analysis. In the case of any inconsistencies during the data analysis, we reached an agreement by referring to the original study. We assessed the risk of bias using the Cochrane Collaboration’s tool [22]. The risk of bias is shown in Additional file 2: Figure S2 and Additional file 3: Figure S3. We performed six analyses to compare the effect of SCT vs. control on LVEF, NYHA class, LVEDV, LVESV, CCS grade, and mortality.

### Publication bias and sensitivity analysis

We analyzed publication bias using both Begg’s rank correlation test and Egger’s linear regression method (Additional file 4: Figure S4) [23]. Each study was individually deleted to assess the effect of the individual data set on the pooled RRs. We then removed the articles that exhibited high heterogeneity. This sensitivity analysis improved the statistical strength of our results.

## Results

### Selected studies and characteristics

**Fig. 1:**
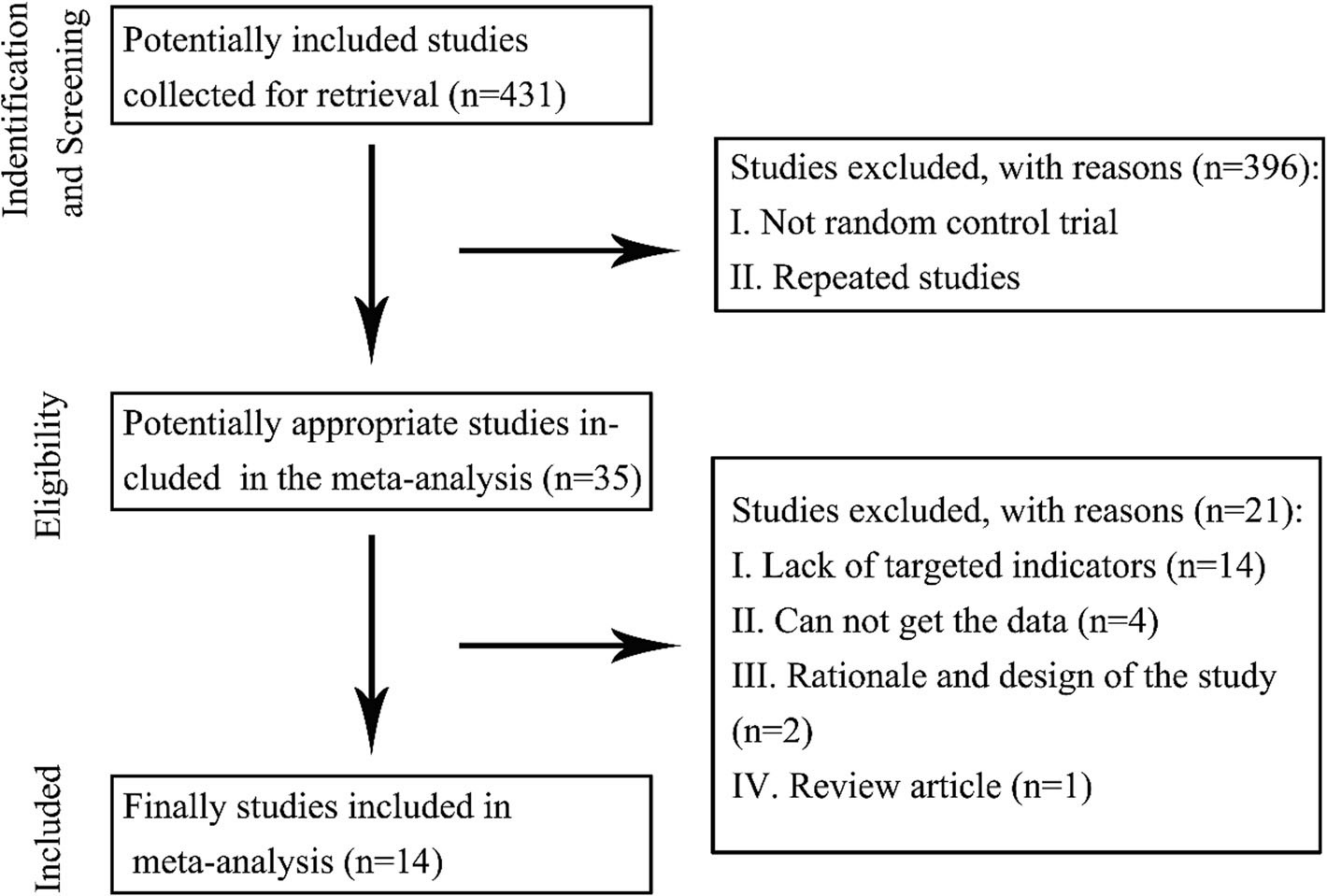
Characteristics of studies evaluating the effect of stem cell transplantation for the treatment of patients with heart failure

We identified 431 reports after a search of all the potential databases. Excluding studies that were not randomized clinical trials resulted in only 35 studies requiring further review. Following a screen of the titles, abstracts, and full texts, we found 14 eligible randomized clinical trials (Fig. 1) [2, 3, 6–8, 11–13, 24–29]. The course of treatment ranged from 2 to 60 months. The detailed characteristics of the studies that evaluated the effects of stem cell transplantation for patients with ischemic heart failure are summarized in Table 1. No evident risk of bias existed in this study.

**Table 1:** Characteristics of patients in the included studies

Author, year	Number	Cell type	Cell dose	Course of treatment (months)
Amit N Patel, 2016 [24]	66	Ixmyelocel-T+BMA	0.8 ml	12
	60	Placebo+BMA	0.8 ml	12
Amit N. Patel, 2005 [11]	10	Stem cells (CD34+)	250 ml	6
	10	Own plasma	30 ml	6
Amit N. Patel, 2015 [12]	24	BMAC infusion	240 ml	12
	6	Medical standard of care	–	12
Anders Bruun Mathiasen, 2015 [13]	40	MSCs	0.2 ml	6
	20	PBS	0.2 ml	6
Emerson C. Perin, 2011 [6]	20	ABMMNC	3 ml	6
	10	Simulated mock injection	–	6
Emerson C. Perin, 2012 [2]	10	ALDHbr	3 ml	6
	10	5% Albumin	3 ml	6
Emerson C. Perin, 2003 [7]	14	ABMMNC	50 ml	2
	7	Placebo	–	4
Jozef Bartunek, 2013 [3]	32	Bone marrow stem cells	50 × 10^6^	24
	15	Standard of care	–	24
Shengshou Hu, 2011 [8]	31	CABG+BMMNC	60 ml	6
	29	CABG	10 ml	6
Zhi Qi, 2015 [25]	24	CABG+BMMNC	60 ml	12
	18	CABG	10 ml	12
Evgeny Pokushalov, 2010 [26]	49	ABMMNC+Medical therapy	41 ± 16 × 10^6^	12
	31	Medical therapy	–	
Nabil Dib, 2009 [27]	12	AMT+MMT	2.5 × 10^7^	12
	11	MMT	–	
Philippe Menasché, 2008 [29]	30	Myoblast	8 × 10^8^	6
	34	Placebo	–	
Qiang Zhao, 2008 [28]	18	BMMNC	An average of 6.59 × 10^8^ ± 5.12 × 10^8^	6
	18	Saline	–	

*BMA* bone marrow aspirate, *BMAC* bone marrow aspirate concentrate, *MSCs* mesenchymal stromal cells, *PBS* phosphate buffer saline, *BMC* bone marrow cell, *ABMMNC* autologous bone marrow mononuclear cell, *ALDHbr* aldehyde dehydrogenase-bright, *MPCs* mesenchymal precursor cells, *CABG* coronary artery bypass graft, *BMMNC* bone marrow mononuclear cell, *AMT* autologous myoblast transplant, *MMT* maximal medical therapy

### Quantitative data synthesis

#### NYHA class

**Fig. 2:**
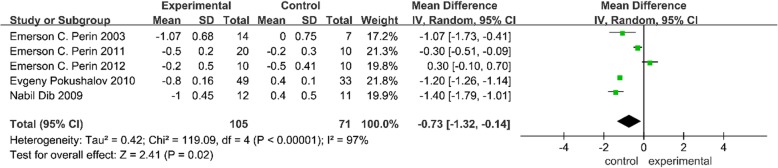
Forest plot of randomized controlled trials comparing the effect of SCT versus control on LVEF

Five trials provided outcomes of the NYHA class [2, 6, 7, 26, 27]. As shown in Fig. 2, a significant amount of heterogeneity (chi-square = 119.09, df = 4, *P* < 0.00001; *I*^2^ = 78%) existed across the trials, and the heterogeneity did not change greatly after omitting each trial one at a time. Therefore, a random effects model was selected for the analysis. The meta-analysis showed that compared with the control group, SCT lowered the NYHA class (MD = − 0.73, 95% CI − 1.32 to − 0.14, *P* < 0.05).

#### LVEF

**Fig. 3:**
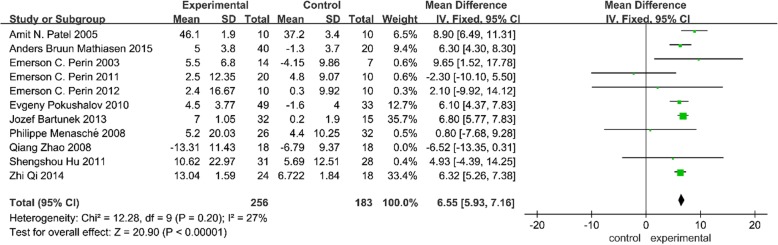
Forest plot of randomized controlled trials comparing the effect of SCT versus control on the NYHA class

Eleven trials reported a change in LVEF [2, 6–8, 11, 13, 25, 26, 28, 29]. As shown in Fig. 3, a trial reported by Zhao et al. was removed for its significant heterogeneity [28]. The heterogeneity of the remaining studies was acceptable (chi-square = 12.28, df = 9, *P* = 0.20; *I*^2^ = 27%); thus, a fixed effects model was used for the analysis across the trials. The MD value and the 95% CI showed a significant increase in LVEF (MD = 6.55, 95% CI 5.93 to 7.16, *P* < 0.05) in the SCT group compared with the control group.

#### LVEDV and LVESV

**Fig. 4:**
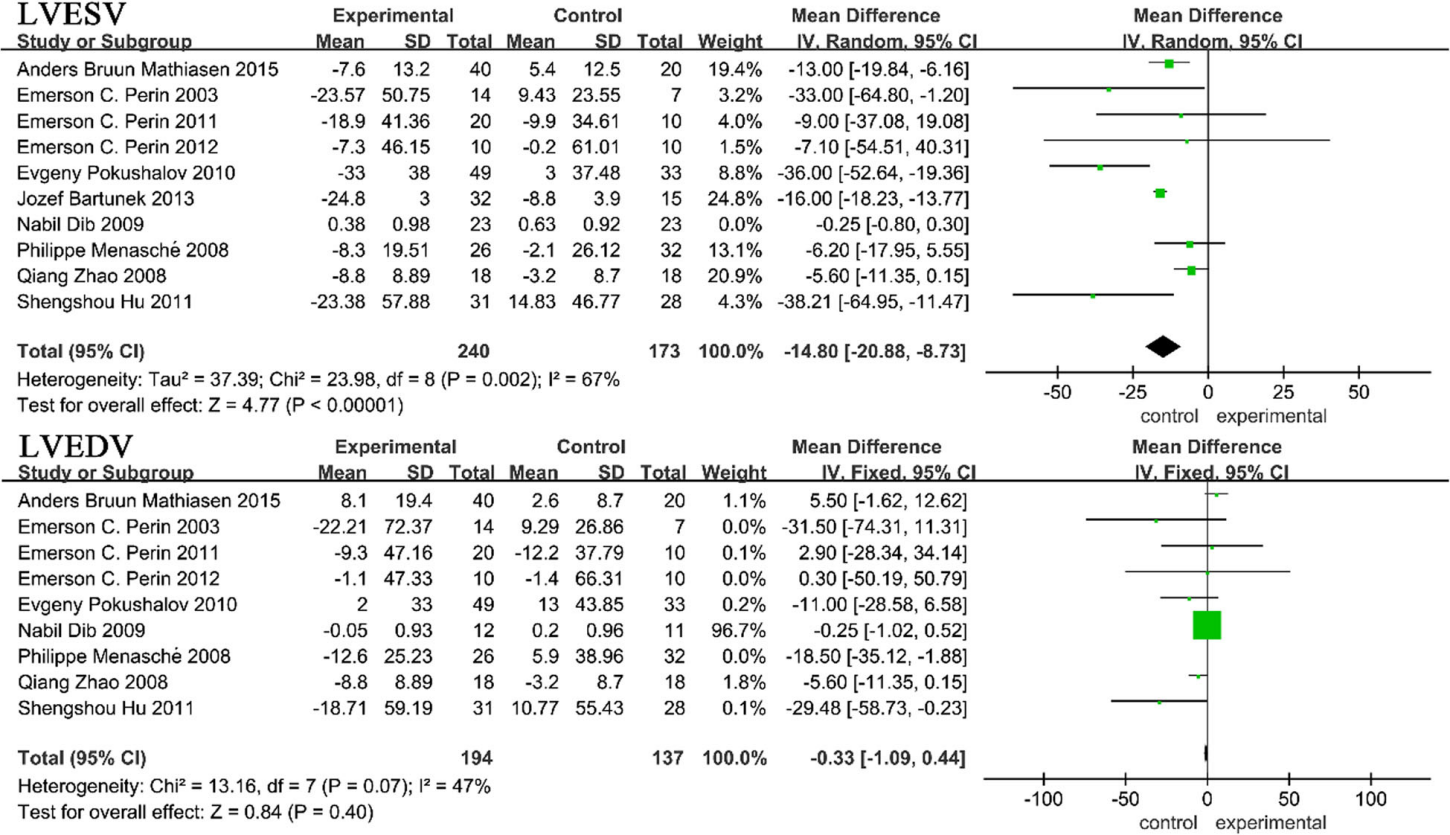
Forest plot of randomized controlled trials comparing the effect of SCT versus control on LVESV and LVEDV

We also evaluated the left ventricular volumes (LVESV and LVEDV). Ten trials reported LVESV values [2, 3, 6–8, 13, 26–29] and nine trials reported LVEDV values [2, 6–8, 13, 26–29]. We found a significant heterogeneity in both analyses. When the trial by Dib et al. was removed from the LVESV analysis, the heterogeneity changed greatly (with *I*^2^ ranging from 96 to 67%) but was still extensive; thus, a random effects model was used [27]. After removing the trial by Menasché et al. from the LVEDV analysis, the heterogeneity was moderate; thus, a fixed effects model was used [29]. As shown in the upper section of Fig. 4, a significantly lower LVESV (MD = − 14.80, 95% CI − 20.88 to − 8.73, *P* < 0.05) was found in patients who underwent SCT compared with those who did not. However, there was no statistically significant change in LVEDV (MD = − 0.33, 95% CI − 1.09 to 0.44, *P* > 0.05) between the two groups (lower section of Fig. 4).

#### CCS grade

**Fig. 5:**
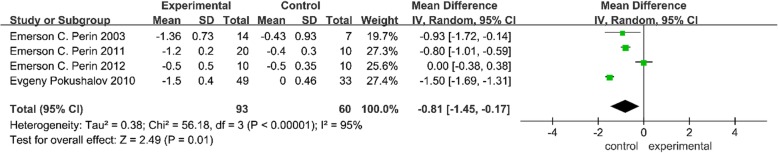
Forest plot of randomized controlled trials comparing the effect of SCT versus control on the CCS grade

Four trials reported the CCS grade (Fig. 5) [2, 6, 7, 26]. An extensive heterogeneity existed in the analysis. The heterogeneity did not change greatly after omitting each trial one at a time. Therefore, a random effects model was selected for the analysis. The pooled estimate indicated that SCT significantly reduced the CCS grade compared with the control group (MD = − 0.81, 95% CI − 1.45 to − 0.17, *P* < 0.05).

### Effect of SCT on mortality

**Fig. 6:**
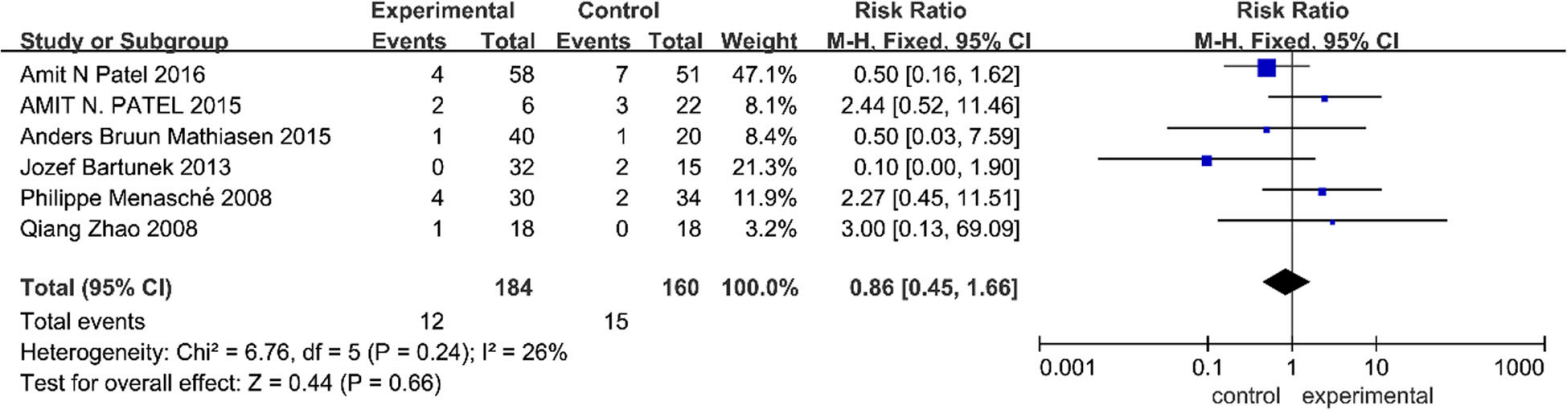
Forest plot of randomized controlled trials comparing the effect of SCT versus control on mortality

Six trials reported outcomes of mortality [3, 12, 13, 24, 28, 29]. No significant heterogeneity existed in this analysis; thus, a fixed effects model was used. As shown in Fig. 6, the pooled estimate indicated that SCT in patients with IHF had no effect on mortality (RR = 0.86, 95% CI 0.45 to 1.66, *P* = 0.66) compared with the control group.

## Discussion

The main symptom of ischemic heart disease is a heart attack or myocardial infarction, which is called an acute myocardial infarction (AMI) when an atherosclerotic plaque ruptures into a coronary artery. Fisher et al. found that stem cell therapy lacks enough evidence for AMI patients; however, more and more medical centers regard SCT as a promising treatment for IHF, which is caused by myocardial remodeling after AMI [30]. Meanwhile, the safety and clinical efficacy of SCT as a treatment for IHF is controversial and requires further evaluation in clinical trials [2, 6, 12, 13]. In the present study, we systematically reviewed and produced a comprehensive meta-analysis of SCT for the treatment of IHF. Our analysis included 14 randomized clinical trials and a total of 669 participants [2, 3, 6–8, 11–13, 24–29]. The included trials recruited patients who had been diagnosed with IHF, and they compared the active intervention (SCT) with a placebo or control group. This study demonstrated the following: (i) SCT was associated with a significant improvement in LVEF, with no effect on mortality, (ii) SCT significantly and moderately reduced LVESV, but not LVEDV, and (iii) IHF symptoms, as indicated by the NYHA functional class and CCS grade, significantly decreased with SCT. Overall, SCT has been shown to be safe as a treatment for IHF with no increase in mortality. Meanwhile, SCT has also shown effectiveness for the improvement of LVEF and the reduction of symptoms (CCS grade and NYHA class).

In our review of different randomized clinical trials, we first found that they reported similar effects, although various parameters differed between the studies, including follow-up time, the number of patients, and cell type that was transplanted to the left anterior descending artery. Most of the included studies used bone marrow mononuclear cells for transplantation to repair the injured heart myocardium, but clinicians also used ALDHbr cells and Ixmyelocel-T, among other cell types. The majority of randomized clinical trials reported an improved LVEF. However, Hu et al. and Bartunek et al. found a decreasing trend in the performance of patients on the 6-min walk test [3, 8]. Therefore, randomized clinical trials with larger sample sizes must be conducted to confirm the exercise function results. Additionally, nearly all the studies reported that the NYHA class improved following SCT compared to the control group. However, Perin et al. did not observe such a result, which is possibly due to the small number of patients enrolled in their study (*n* = 20) and its subsequent influence on their statistical analysis [2, 6, 7]. The quality of life is of utmost importance. Comorbidities and anxiety/depression disorders are associated with worse health-related quality of life. But we did not report the quality of life for lacking enough data in the RCTs.

Compared with other relevant meta-analyses, we found that Fisher et al., Tian et al., and Wen et al. assessed LVEF, LVESV, LVEDV, and adverse events in ischemic heart disease patients. These authors found that intramyocardial BMC treatment contributed to an improvement in left ventricular dysfunction. They further found a reduction in LVESV and a decreasing trend in LVEDV, which partly supports the findings of our meta-analysis in regard to LVEF and LVESV [18, 31, 32]. Fisher et al. also reported that cell therapy reduced the incidence of long-term mortality, which is very important for the persistent treatment of IHF [18]. Gyöngyösi et al. revealed that SCT conferred no benefits for patients with IHF, based on clinical events or changes in left ventricular function, which was different from our study [33]. Fisher et al. reported that SCT could reduce mortality, although they included studies that were not randomized clinical trials. Among the other studies, the authors found that the NYHA class and LVEF significantly improved within 12 months of treatment, while LVEF after 12 months did not significantly increase, other than in our meta-analysis [34]. Furthermore, we analyzed LVEDV and LVESV, which were observed to be improved after SCT. Both our study and that by Fisher et al. reported that SCT, regardless of stem cell type, could improve LVEF, but our study showed no effect on mortality as we know mortality is an important indicator in stem cell therapy. Cheng et al. revealed that SCT did not improve LVEF, but increased the 6-min walk distance, reduced the incidence of NYHA functional class deterioration, and improved the MLHF score. The authors also found that SCT did not change the mortality; these results were consistent with our study regarding the NYHA class and mortality, but inconsistent in regard to LVEF [35].

Although we used DerSimonian and Laird’s random effects model while pooling the individual studies to compensate for statistical heterogeneity, some heterogeneity still existed [36]. One indicator (CCS grade) was assessed in a small sample size. Therefore, potential publication bias could not be excluded. Standardized quality of life and major adverse events were also needed to be further analyzed regarding as secondary indicator. Meanwhile, this meta-analysis was also restricted to patients with ischemic heart failure events occurring in independent populations. Despite these limitations, our meta-analysis provided evidence that supports stem cell transplantation as an effective treatment for patients with ischemic heart failure. Furthermore, there were several strengths of the present study. Our results indicated that there was a paltry risk of bias in the study, thereby suggesting that the results are relatively reliable. Only randomized clinical trials were included, to ensure a high quality of the studies. To the best of our knowledge, our study provides new insights into SCT for treating patients with ischemic heart failure.

## Conclusion

In conclusion, this meta-analysis suggests that stem cell transplantation is a safe and effective treatment option for patients with IHF since SCT resulted in a reduction in the NYHA class, CCS grade, and LVESV, as well as an increase in LVEF, but did not affect mortality. More well-designed randomized clinical trials are required to confirm these results.

## Additional files

Additional file 1: **Figure S1.** The PRISMA checklist for this study. (DOCX 541 kb)

Additional file 2: **Figure S2.** Risk of bias graph: overview of authors’ judgements about each risk of bias item for each included study. (DOCX 85 kb)

Additional file 3: **Figure S3.** Risk of bias summary: review of authors’ judgements about each risk of bias item for each included study. (DOCX 289 kb)

Additional file 4: **Figure S4.** Funnel plot of the studies included in the meta-analysis, which was used to test for publication bias. (DOCX 74 kb)

## Abbreviations

BMMNCs: Bone marrow mononuclear cells
CCS: Canadian Cardiovascular Society
CI: Confidence interval
IHD: Ischemic heart disease
IHF: Ischemic heart failure
LVEDV: Left ventricular end-diastolic volume
LVEF: Left ventricular ejection fraction
LVESV: Left ventricular end-systolic volume
MD: Mean difference
M-H: Mantel-Haenszel
NYHA: New York Heart Association
PICO: Population, intervention, comparison, and outcomes
PRISMA-IPD: Preferred Reporting Items for Systematic Reviews and Meta-Analyses of individual participant data
RCT: Randomized controlled trial
RR: Relative risk
SCT: Stem cell transplantation
SD: Standard deviation
WMD: Weighted mean difference
