# Supplementary figures and images for: Effect of stem cell transplantation on patients with ischemic heart failure: a systematic review and meta-analysis of randomized controlled trials
Source: Stem Cell Res Ther. 2019 Apr 18;10:125. doi: 10.1186/s13287-019-1214-0 (PMC6472092; doi:10.1186/s13287-019-1214-0)

**
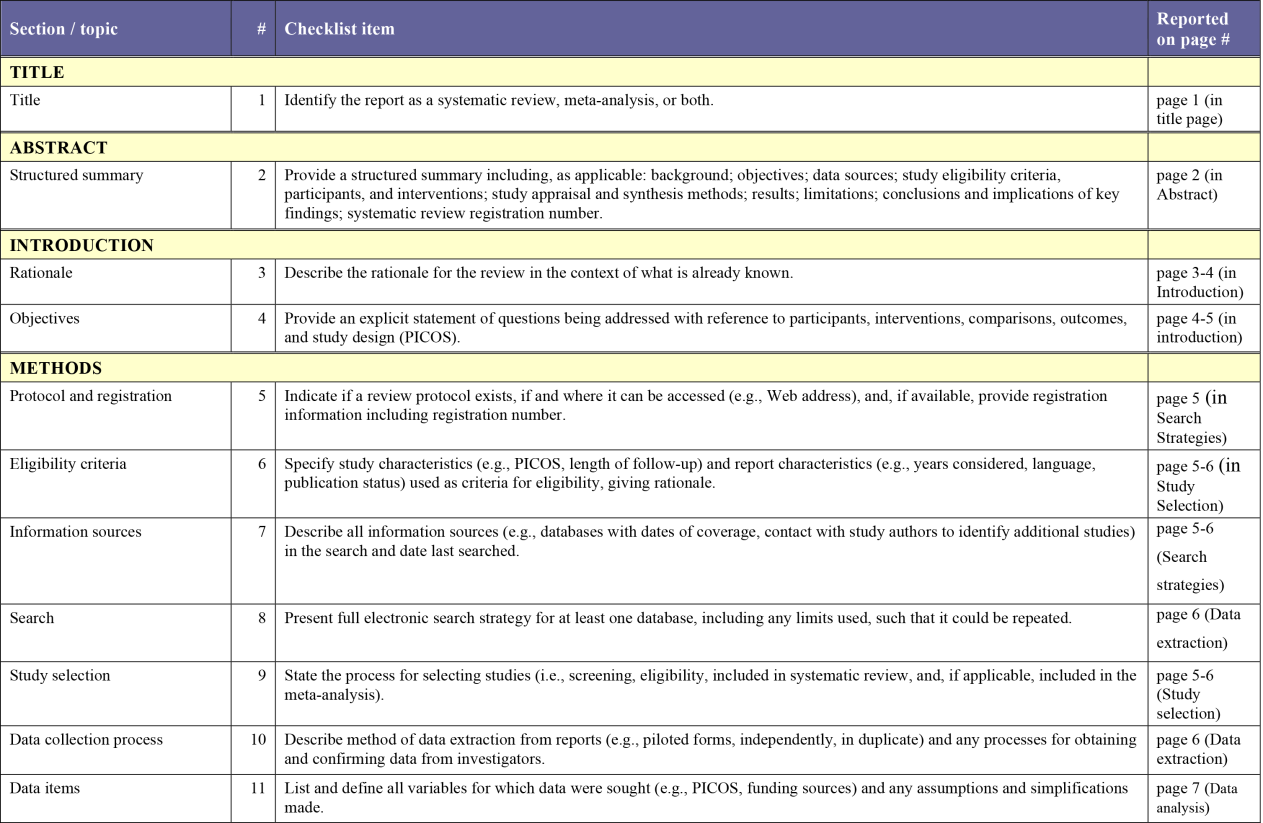
**

**
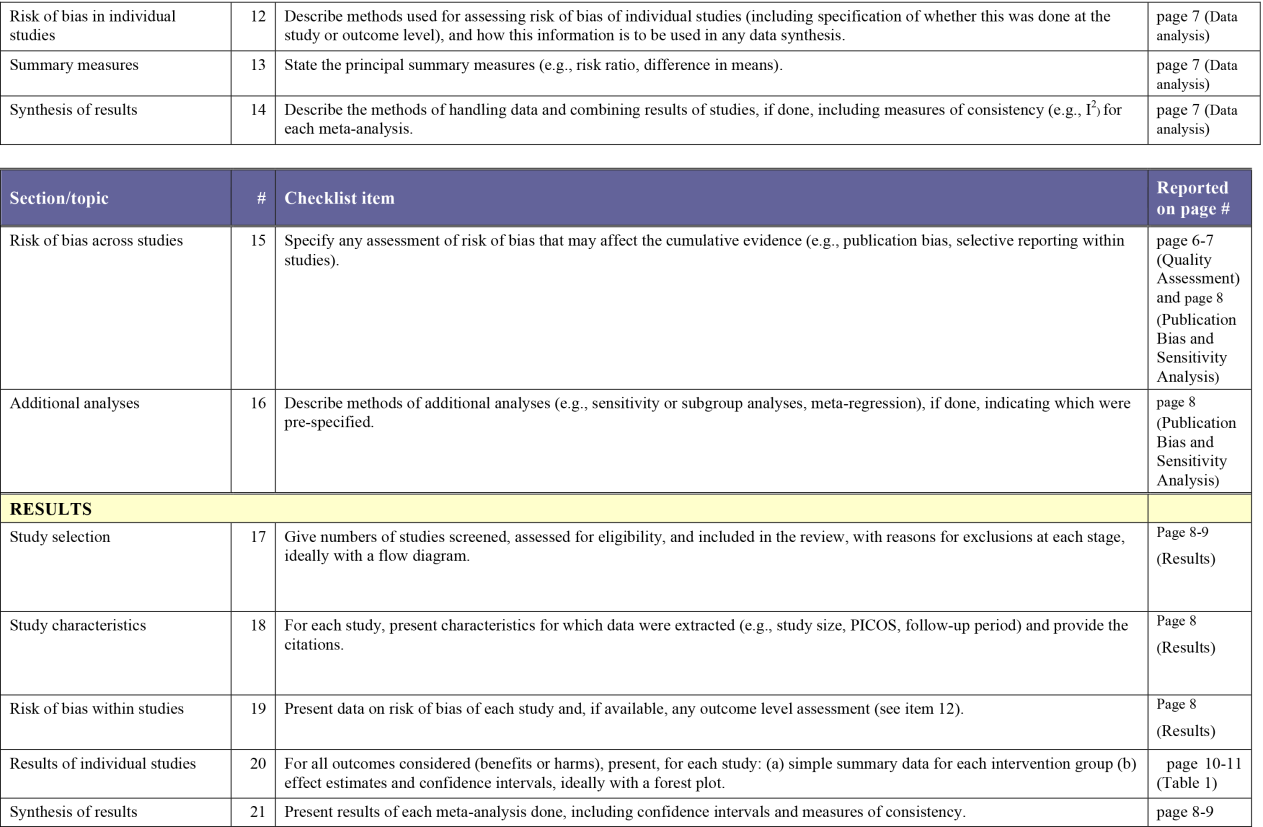
**

**
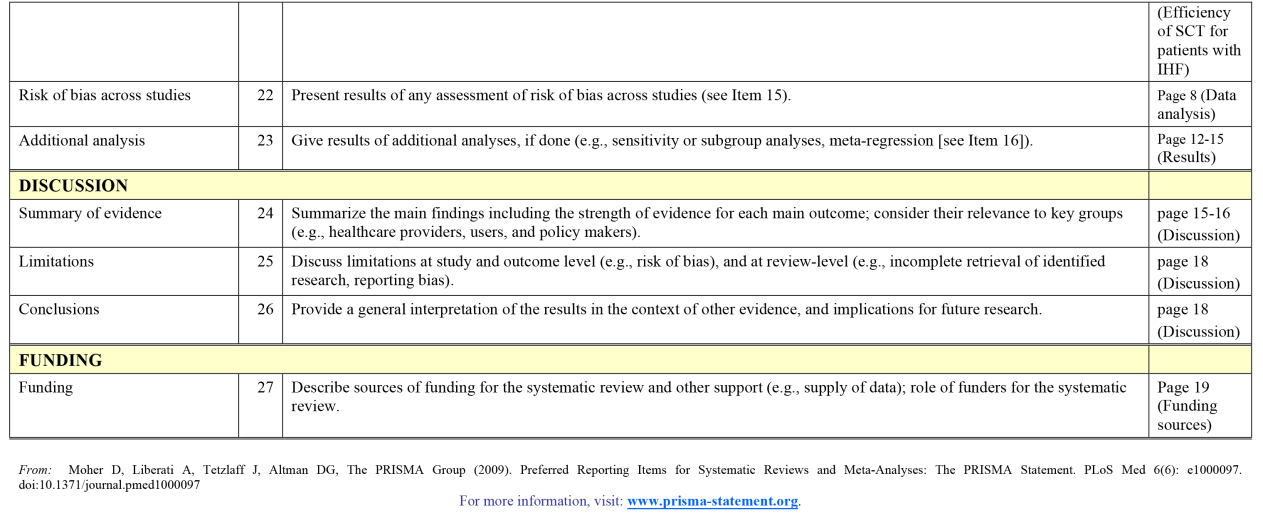
**

Supplement: Supplementary file 1 — Figure S1. The PRISMA checklist for this study. (DOCX 541 kb) [file 13287_2019_1214_MOESM1_ESM.docx]

**
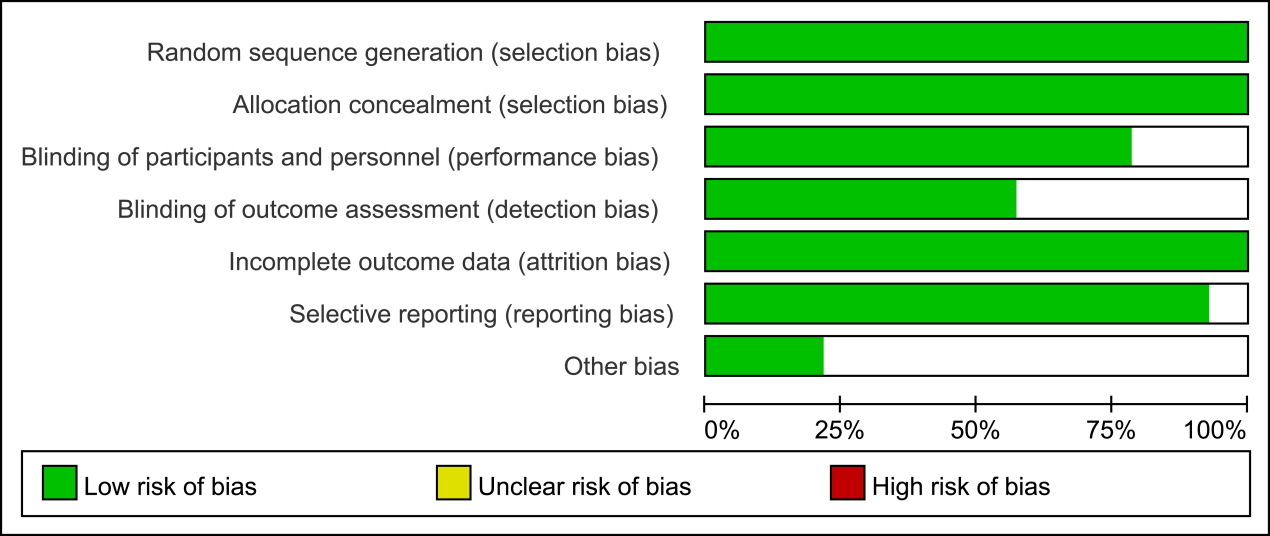
**

Supplement: Supplementary file 2 — Figure S2. Risk of bias graph: overview of authors’ judgements about each risk of bias item for each included study. (DOCX 85 kb) [file 13287_2019_1214_MOESM2_ESM.docx]

**
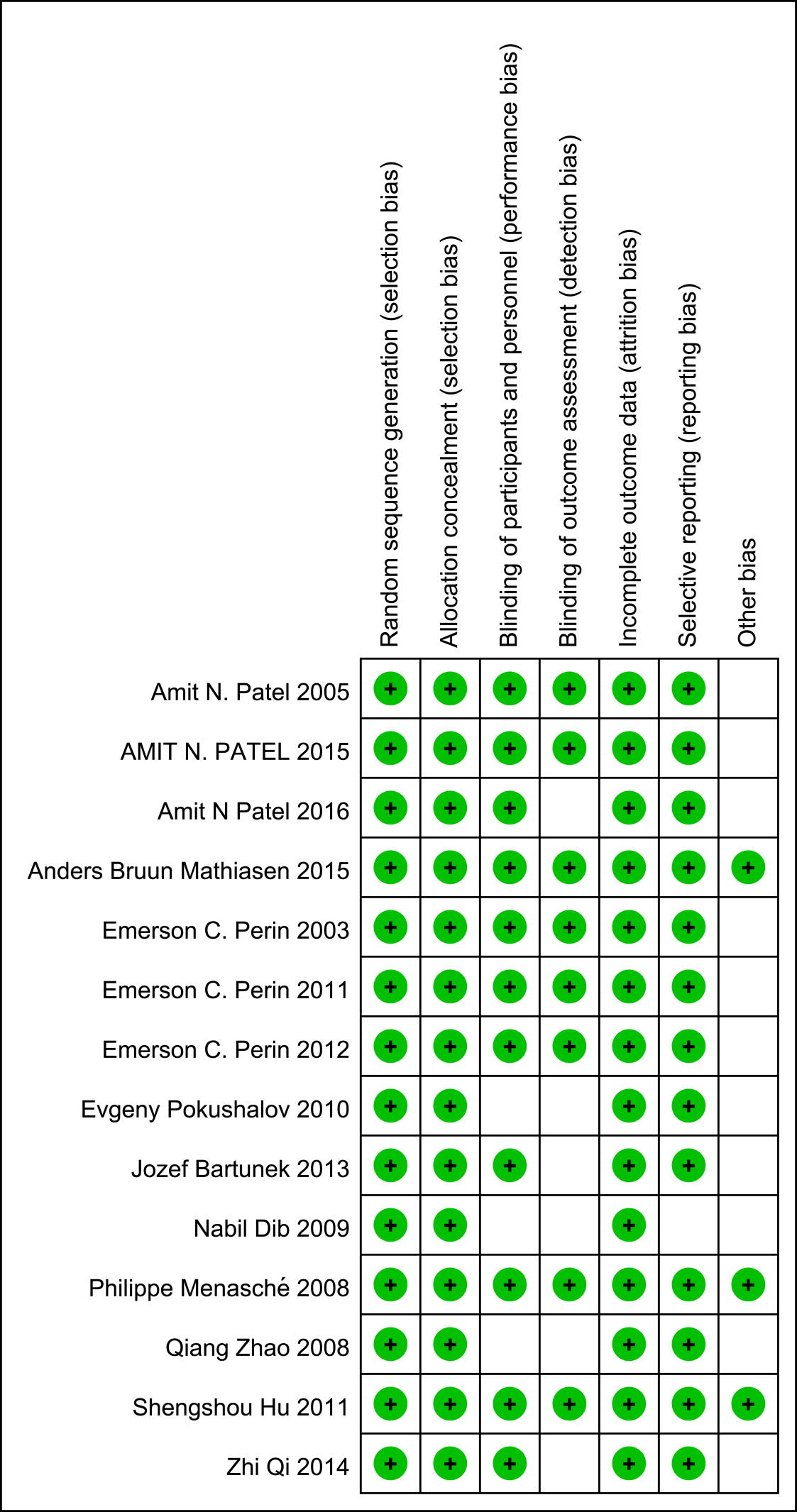
**

Supplement: Supplementary file 3 — Figure S3. Risk of bias summary: review of authors’ judgements about each risk of bias item for each included study. (DOCX 289 kb) [file 13287_2019_1214_MOESM3_ESM.docx]

**
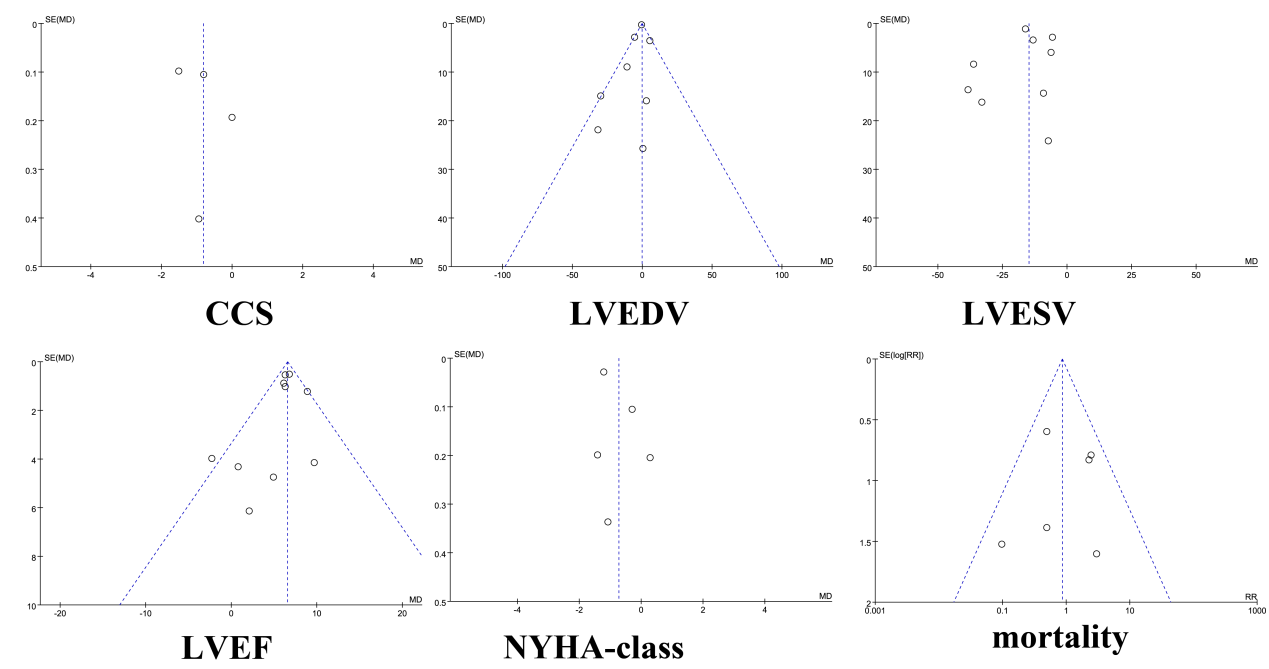
**

Supplement: Supplementary file 4 — Figure S4. Funnel plot of the studies included in the meta-analysis, which was used to test for publication bias. (DOCX 74 kb) [file 13287_2019_1214_MOESM4_ESM.docx]
